# The Multiple Biological Functions of Dipeptidyl Peptidase-4 in Bone Metabolism

**DOI:** 10.3389/fendo.2022.856954

**Published:** 2022-05-02

**Authors:** Qiu Yang, Bing Fu, Dan Luo, Haibo Wang, Hongyi Cao, Xiang Chen, Li Tian, Xijie Yu

**Affiliations:** ^1^ Department of Endocrinology and Metabolism, Laboratory of Endocrinology and Metabolism, West China Hospital, Sichuan University, Chengdu, China; ^2^ Department of Endocrinology and Metabolism, Chengdu Fifth People’s Hospital, Chengdu, China; ^3^ Department of Medical Imaging, Chengdu Fifth People’s Hospital, Chengdu, China; ^4^ Department of General Surgery, Chengdu Fifth People’s Hospital, Chengdu, China

**Keywords:** dipeptidyl peptidase-4, bone metabolism, osteoimmunology, adipokines, bone microenvironment, cytokines

## Abstract

Dipeptidyl peptidase-4 (DPP4) is a ubiquitously occurring protease involved in various physiological and pathological processes ranging from glucose homeostasis, immunoregulation, inflammation to tumorigenesis. Recently, the benefits of DPP4 inhibitors as novel hypoglycemic agents on bone metabolism have attracted extensive attraction in many studies, indicating that DPP4 inhibitors may regulate bone homeostasis. The effects of DPP4 on bone metabolism are still unclear. This paper thoroughly reviews the potential mechanisms of DPP4 for interaction with adipokines, bone cells, bone immune cells, and cytokines in skeleton system. This literature review shows that the increased DPP4 activity may indirectly promote bone resorption and inhibit bone formation, increasing the risk of osteoporosis. Thus, bone metabolic balance can be improved by decreasing DPP4 activities. The substantial evidence collected and analyzed in this review supports this implication.

## 1 Introduction

The human dipeptidyl peptidase-4 (DPP4) is a cell surface glycoprotein widely expressed in various tissue compartments, including the lymph gland, the biliary tract, the kidneys, the liver, the intestinal system, and bone marrow (1, 2). It is a member of the serine peptidase/prolyl oligopeptidase family, which possesses enzyme functions and selectively cleaves off the penultimate alanine, proline, or serine in the N-terminus start site. Confirmed substrates for DPP4 *in vivo* included many incretins and cytokines (3, 4). In addition, DPP4 was previously known as the T-cell activation antigen cluster of differentiation-26 (CD-26). It has been known for playing a complex role in non-enzyme–dependent functions by acting as costimulatory proteins in immune cells such as T cells, monocytes, and dendritic cells. Functionally, it is involved in multiple physiological processes and pathologies ranging from glucose homeostasis, immunoregulation, inflammation to tumorigenesis.

Over the past decade, it has aroused public concern for its pleiotropic actions on bone metabolism. First, as a new oral hypoglycemic drug, DPP4 inhibitors might promote bone formation and inhibit bone absorption in addition to possessing practical hypoglycemic effects, reducing the risk of osteoporosis and fracture (5–9). Second, cells in the bone microenvironment, such as osteoclasts, bone marrow adipose tissue (BMAT), and immune cells, would secrete DPP4 (10–12). Third, the substrates of DPP4 are distributed on bone cells, bone immune cells, and cytokines in the skeleton system (3, 4). Therefore, it can be speculated that DPP4 may directly or indirectly regulate bone metabolism. This study reviews the regulation mechanisms of DPP4 on bone energy metabolism, bone immunity, and bone remodeling, aiming to provide a new target for the treatment of osteoporosis.

## 2 Structure and Functions of DPP4

### 2.1 Molecular Properties and Forms

**Figure 1:**
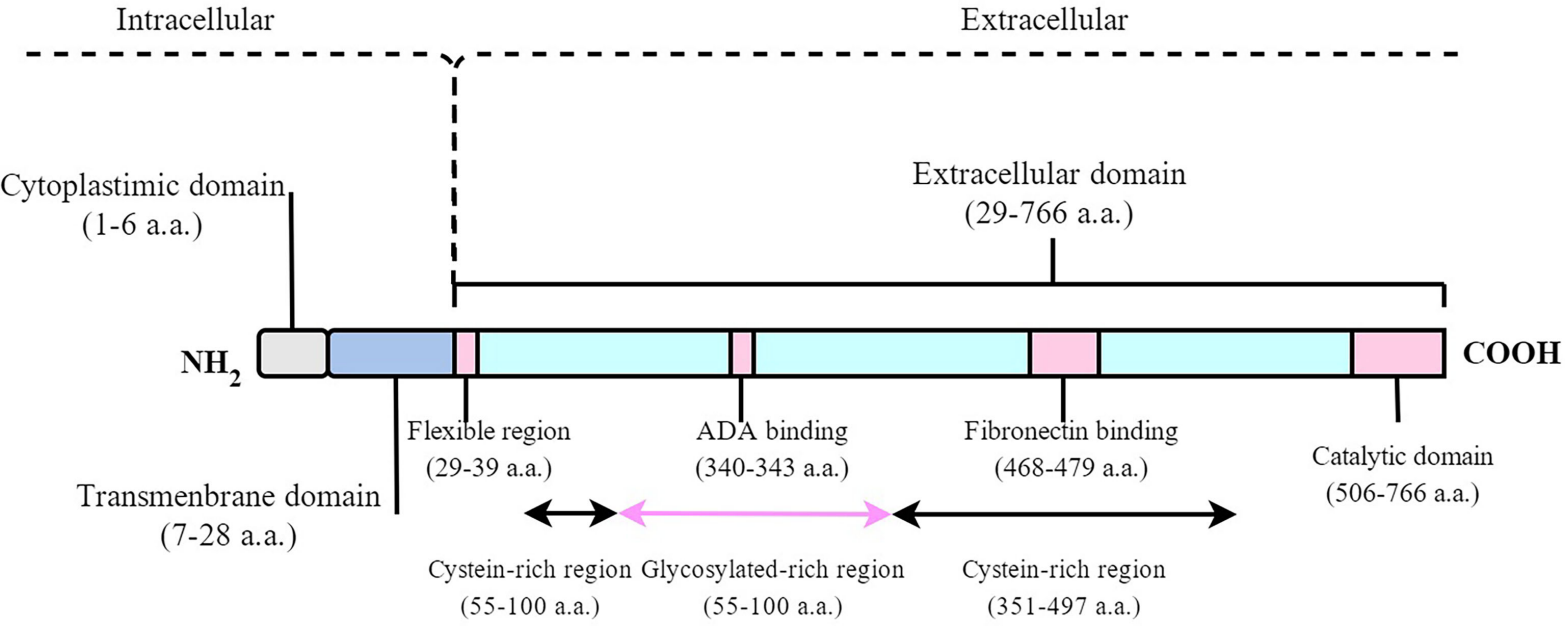
Schematic diagram of the human DPP4 molecule. mDPP4 consists of three domains with 766 residues, including a 6-amino acid N-terminal cytoplasmic domain, a 22-residue hydrophobic transmembrane domain, and an extracellular domain. sDPP4 contains 727 residues without cytoplasmic domain, transmembrane domain, and flexible region.

DPP4/CD26 is a dimeric 240-kDa glycoprotein composed of two 120-kDa subunits and coded by a gene on chromosome 2q24.3 (13). DPP4 can be divided into two different forms: either as a soluble form (sDPP4) in circulation or a membrane-bound form (mDPP4) anchored to a cellular surface (14). mDPP4 consists of three domains with 766 residues, including a six–amino acid N-terminal cytoplasmic domain, a 22‐residue hydrophobic transmembrane domain, and an extracellular domain. The extracellular domain includes the cystein-rich region, glycosylated-rich region, adenosine deaminase (ADA)–binding region (340–343 a.a.), fibronectin-binding region (469– 479 a.a.), and c-terminal DPP4 catalytic sites (507–766 a.a. with active catalytic sites at 630, 708, and 740) (**Figure 1**). mDPP4 is widely expressed on the surface of epithelial, endothelial, stromal, preadipocytes, mature adipocytes, embryonic stem cells, hematopoietic stem cells, hematopoietic progenitor cells, and immune cells (11, 15–23) in different tissues and organs, such as the liver, gut, adipose tissue, and bone marrow (1, 2). sDPP4 contains 727 residues without a cytoplasmic domain, a transmembrane domain, and flexible region (24). sDPP4 is considered as the hydrolyzed form of mDPP4, which is widely present in serum, saliva, cerebrospinal, seminal fluid, and bile and has fully enzymatically active (25, 26). The protease responsible for generating sDPP4 is unclear. The different physiological function between sDPP4 and mDPP4 is not well known.

### 2.2 Multifunctional Properties

DPP4 is a member of the serine peptidase/prolyl oligopeptidase family, which selectively cleaves the penultimate alanine, proline, or serine in the N-terminus start site, including incretins and cytokines. Glucagon-like peptide-1 (GLP-1), glucagon-like peptide-2 (GLP-2), glucose-dependent insulinotropic peptide (GIP) (3), peptide YY (PYY), and neuropeptide Y (NPY) (4) are the most widely studied incretins. Besides the factors mentioned above, Ou et al. (2) identified many proteins as cleaved proteins for DPP4 from the National Center for Biotechnology Information database and Universal Protein Resource.

In addition, DPP4 plays a complex role in classical enzyme functions as well as various subcellular localization and non-enzyme-dependent functions. DPP4 performs its non-proteolytic functions as receptor or costimulatory protein in the fields of immunology. It is a receptor of ADA (27, 28), which plays an important role in immune regulation as the key enzyme for adenosine decomposition. The formation of DPP4-ADA complex reduces local concentration of adenosine, alleviating the inhibitory effect of high concentration adenosine on T lymphocyte proliferation and activation (29). The DPP4-ADA complex promotes the differentiation of primary T lymphocytes into helper T cells and memory T cells (30). Meanwhile, DPP4-ADA complex also plays a costimulatory role in immunological synapse between dendritic cells and CD4^+^ T cells, enhancing inflammatory response by inducing interleukin-6 (IL-6), interferon-γ (INF-γ), and tumor necrosis factor-α (TNF-α) secretion (31, 32). Furthermore, DPP4 also has costimulatory function by binding to CXC chemokine receptor-4 (CXCR4) (33), mannose 6 phosphate/insulin-like growth factor II receptor (M6P/IGF-IIR) (34), TCR/CD3 (30), CD45 (35), caveolin-1 (36), CARMA1 (37), and fibronectin III (38, 39). This evidence suggests the immunomodulation function of DPP4 in different signaling process.

## 3 Scientific Evidence of the Relationship Between DPP4 and Bone Metabolism

### 3.1 The Relationship Between Serum DPP4 Activity and BMD

A study of 744 postmenopausal Chinese women with normal glucose tolerance showed significant lower in lumbar and femoral neck bone marrow density (BMD) in the quartile with the highest DPP4 activity (40). However, a 13-year follow-up study (41) of 1,536 community-based elderly adults revealed that there was no significant association between basal DPP4 activity and hip BMD, lumbar spine BMD, or incidence of hip fractures. A cross-sectional study with 147 newly diagnosed T2D showed that elevated sDPP4 activity was positively associated with the risk of osteoporosis/osteopenia and fracture (42). However, a study (43) of 204 male Japanese diabetics showed no correlation between DPP4 levels and BMD in the lumbar spine or femoral neck. The relationship between DPP4 and BMD has been reported to depend on BMI. Durinx et al. (44) demonstrated that DPP4 activity was negatively correlated with spine BMD in obese postmenopausal women, which was not found in non-obese postmenopausal women. From the evidence mentioned above, it prompted that the correlation between DPP4 activity and bone metabolism might be influenced by aging and metabolic status. Durinx et al. (44) studied 481 healthy subjects aged between 19 and 61 years and revealed an age related decline in DPP4 activity. Indeed, previous studies have showed that DPP4 was a novel adipokine (45) released from immune cells, adipose cells, and bone marrow cells, which correspondingly indicated that sDPP4 concentrations and activity were linked to obesity, metabolic syndrome, T2D, and inflammatory diseases (10, 46, 47). When evaluating DPP4 activity and bone metabolism, stratified population analysis has to be taken into account.

### 3.2 The Impact of DPP4 Inhibitors on Bone Metabolism in the Pre-Clinical and Clinical Studies

A significant number of pre-clinical studies have shown a beneficial impact on osteogenesis for DPP4 inhibitors. Intraperitoneal injections of sitagliptin for 9 days significantly increased the osteogenic progenitors while decreased the adipogenic progenitors in non-fractured tibiae (5). Sitagliptin would increase the BMD of mice with a high-fat diet (6). In addition, the negligible BMD loss was observed in OVX non-diabetic adult rats treated with the higher sitagliptin doses, compared with thiazolidinediones or placebo for 12 weeks (7). In addition, DPP4 inhibitors showed the function of pro-osteoblastogenesis and anti-osteoclastogenesis. Anagliptin significantly promoted the osteoblastic differentiation of MC3T3-E1 cells and increased matrix deposition and mineralization (5). Linagliptin downregulated LPS-induced osteoclast formation and bone resorption (8). Nishida et al. (9) showed that the humanized anti-CD26 monoclonal antibody (hu-CD26mAb) inhibited osteoclast precursor differentiation.

Data from animal studies suggested that DPP4 inhibitors (sitagliptin, saxagliptin, vildagliptin, and linagliptin) have an anabolic effect on bone. However, clinical studies of DPP4 inhibitors remained controversial (5). Dombrowski et al. (48) found that DPP4 inhibitors were associated with a decrease in the risk of osteoporotic fracture by matching 4,160 patients with DPP4 inhibitors to never users. A meta-analysis of 28 trials showed 40% reduction in fracture risk with the treatment of DPP4 inhibitors (11,880 T2D) compared to placebo or other treatments (9,175 T2D) in a duration of 24 weeks (49). A retrospective population–based (49) cohort study of T2D (N = 216,816) revealed that DPP4 inhibitor usage was not associated with change in fracture risk compared to controls and other non-insulin anti-diabetic drug usage. Since the average duration of 1.3 years might have been too short to show association with fracture risk.

In contrast, a *post hoc* pooled analysis of 20 randomized controlled studies in 9,156 patients with T2D revealed that the incidence rate for bone fracture was higher with saxagliptin versus control (50). Different study designs may explain the different findings because the studies included in the pooled analyses took higher doses than approved saxagliptin doses (2.5 mg/day) with almost up to 5 or 10 mg/day or even 100 mg/day. Meanwhile, another network meta-analysis (51) including 117 RCTs of 221,364 T2D, compared anti-diabetic drugs head-to-head. Specifically, trelagliptin, omarigliptin, sitagliptin, vildagliptin, and saxagliptin may increase the risk of bone fracture, whereas others (linagliptin and alogliptin) may show benefits. It indicated that fracture risk was independent of age, sex distribution, and the duration of exposure to anti-diabetic drugs.

With aging, diabetes, obesity, and menopause status were risk factors for osteoporotic and bone fractures, thus it is hard to conclude the association between DPP4 inhibitors and bone metabolism from the current clinical evidence. Further controlling confounding factors, long-term studies are needed to confirm the effect of DPP4 inhibitors on osteoporotic fracture in none-diabetes, non-menopausal, and different levels of obesity population.

## 4 Mechanisms of the Effects of DPP 4 on Bone Metabolism

Some previous studies have tried to prove whether the beneficial effects of DPP4 inhibitors on bone metabolism arise from a direct action on the bone cells. Ishida et al. (52) found that DPP4 inhibitor had no direct impact on the differentiation and proliferation of osteoclast precursors or on the ligand of receptor activator of nuclear factor κB (RANKL) expression in bone marrow stromal cells. Gallagher et al. (53) investigated the effect of a DPP4 inhibitor (MK-0626) on bone metabolism in an animal model of T2D. They revealed that MK-0626 had no adverse effects on bone *in vivo* or no direct effect on osteoblasts *in vitro*. The results also suggested that the effects of DPP4 on bone remodeling might mediate through indirect mechanisms instead of direct impacts.

### 4.1 DPP4 Takes Part in the Bone Metabolism Through a Network of Adipokines

There is an essential relationship between bone remodeling and energy metabolism since bone remodeling requires energy. A great number of observations have shown that adipose could regulate energy metabolism *via* adipokines based on the thesis of adipose-bone interactions (54). In the last decade, BMAT was found to be a kind of fat depot within the marrow niche with different origin, structure, and function.

It was shown that the BMAT is a metabolically active fat depot that plays a role in lipid storage and metabolic homeostasis (45). In addition, BMAT is involved in the bone–fat interaction through a complex network of adipokines such as adiponectin and leptin.

DPP4 is a newly discovered adipokine that originated from mature adipocytes (55–57) including bone marrow adipocytes (10, 11). Mulvihill et al. (11) revealed that bone marrow–derived cells contributed to ~40%–50% of sDPP4 activity in the fasting state by three gene knockout (KO) mice models. In addition, Weivoda et al. (12) utilized denosumab (DMAb) to pharmacologically ablate osteoclasts and RNA sequencing of bone biopsies in postmenopausal women, demonstrating that DPP4 is not only an osteoclast-derived protein but also links bone remodeling to energy metabolism. Thus, it was deducible DPP4 might play an essential role in the adipokines network of bone energy metabolism.

#### 4.1.1 Adiponectin

Adiponectin is mainly from BMAT (58) and secreted from MSC-derived adipocytes (59). Its receptor is expressed on osteoblasts and osteoclasts. Adiponectin would promote the differentiation and activity of osteoblasts and inhibit the differentiation of preosteoclasts and bone resorption (60–62). Evidence had proved a negative correlation between the activity of DPP4 and the circulating level of adiponectin in lean and obese subjects (63). Meanwhile, a systematic review and meta-analysis of randomized controlled trials showed that treatment of DPP4 inhibitors would increase the plasma concentrations of adiponectin (64). Exendin-4, a GLP-1 receptor agonist, increased the expression and secretion of adiponectin *via* the Protein Kinase A (PKA) pathway in 3T3-L1 adipocytes (65). In addition, Piao et al. found a cross-talk between DPP4/GLP-1/GLP-1R and adiponectin/adiponectin receptor in ischemic vascular regeneration under chronic stress conditions (66). From the accumulated evidence, it was speculated that DPP4 might diminish the positive effect of adiponectin on bone mass.

#### 4.1.2 Leptin

Leptin signaling is one of the critical pathways affecting human energy metabolism, and its biological functions are complicated. Leptin has a dual effect on bone tissue, which can either centrally inhibit bone formation by binding to leptin receptors in the hypothalamus or locally promoting bone formation and inhibiting bone resorption by binding to the expressed receptors on the surface of osteoblasts (67, 68). Leptin is not a substrate of DPP4 but has a putative DPP4 truncation site. Anagliptin (DPP4 inhibitor) ameliorated leptin resistance and attenuated food intake and body weight in diet-induced obesity mice (69). A group of Japanese T2D patients was treated with anagliptin and metformin or miglitol for 52 weeks, and reduced leptin concentration was observed (70). However, the exact function of truncated leptin on bone remains unknown.

### 4.2 DPP4 Regulates Bone Remodeling by Binding to Its Substrates Locating in the Skeletal System

DPP4 might indirectly regulate bone remodeling by binding to multiple peptides substrates including incretins, gastrointestinal peptides, and neuropeptides, whose receptors are widely expressed in skeletal system. Gastrointestinal peptides, such as GLP-1, GLP-2, and GIP have been shown to favor bone formation over resorption, whereas NPY and PYY have been shown to have catabolic effects on bone metabolism (**Table 1**).

**Table 1:** Summary of the effects of the gut hormones and neuropeptide on bone metabolism.

Hormone	Receptors	Receptors on bone cells	Effects on bone cells			Effects on bone		
			proliferation	differentiation	apoptosis	Bone formation parameters	Bone resorption parameters	BMD and Bone strength
GLP-1	GLP-1R	BMSCs, primary osteoclasts, osteoblasts	differentiation and proliferation of osteoblastic cell lines (MC3T3-E1, TE-85, and MG-63) ↑	osteogenic differentiation of BMSCs ↑	/	P1NP ↑ ALP ↑	CTX ↓	BMD↑ improved trabecular structure bone strength ↑
GLP-2	GLP-2R	osteoclasts	/	/	/	P1NP ↓	CTX ↓	aBMD↑
GIP	GIPR	osteoblasts, osteoclasts, osteocytes, BMSC	/	/	/	P1NP ↑ALP ↑	CTX ↓	BMD ↑ bone loss ↑
NPY	Y1R	osteoblasts, osteocytes, osteoclasts, chondrocytes	inhibited osteoblast activity by binding to the osteoblastic Y1 receptor				Bone resorption parameters↓	bone mass ↓
PYY	Y1R	osteoblasts	/	/	/	P1NP ↓	/	aBMD ↓ bone mass ↓ bone strength ↓

The symbols ↑ means elevation. The symbols ↓ means reduction.

#### 4.2.1 Anabolic action

GLP-1 is an incretin hormone originating from the distal small intestine and would be degraded at the N-terminus by DPP4 (71–73). The GLP-1 receptor (GLP-1R) is expressed on human bone marrow stem cells (BMSCs) but not on mature osteoblasts (74) and MLO-Y4 osteocytic cell line (75). In human BMSCs, GLP-1 inhibited adipocyteogenesis but promotes bone formation through upregulating osteocalcin (OCN) and osteoprotegerin (OPG) (76–78). Furthermore, GLP-1 and GLP-1R agonists (exendin-4 and liraglutide) are proved to increase the proliferation and differentiation of osteoblasts (79–81). In animal studies, GLP-1R KO mice show decreased bone quality and strength and reduced cortical area (82). In contrast, GLP-1R agonists (liraglutide and exendin-4) increased aBMD and improved trabecular structure and bone strength (83, 84). The mechanisms of GLP-1R–mediated osteogenic action are exerted through a dual role: the cAMP/PKA/b-catenin/T cell factor (TCF) pathway to initiate osteoblast differentiation and the PKA/PI3K/Akt/GSK3b pathway to inhibit ß-catenin degradation and promote its nuclear accumulation in BMSCs, which resulted in the anabolic bone formation (74).

GLP-2 was co-secreted with GLP-1 in the intestine (85) and was deactivated by sDPP4 with a half-life of 5 to 7 min (86). The GLP-2 receptor (GLP-2R) was expressed on osteoclasts (87). Both short-term (14 days) (88) and long-term (4 months) (89) administrations of GLP-2 would reduce bone resorption (CTX) in a dose-dependent manner without influencing bone formation (OCN and P1NP). Four-months of treatment with GLP-2 enhanced hip BMD in postmenopausal women (89).

GIP mainly originates from the proximal small intestine (90) and is degraded by DPP4 with a half-life of about 4 min in human serum (91). The GIP receptor (GIPR) is expressed on BMSCs (92), osteoblasts (93), osteoclasts (94), and osteocytes (93). *In vitro*, stimulating osteoblasts with GIPR would reduce its apoptosis (91) and increase their viability, alkaline phosphatase activity, and type 1 collagen expression (93, 95). *In vivo*, GIPR KO mice exhibit low bone mass (91), altered bone microarchitecture, decreased markers of bone formation (96), and cortical osteopenia due to decreased bone formation and increased bone resorption. In clinical studies, reduced collagen type-1 (CTX-1) and increased procollagen type I N-terminal propeptide (P1NP) were observed in short-term GIP infusion in both healthy humans and patients with type 1 diabetes (97). These results indicate that GIP can uncouple formation and bone resorption and play an anabolic role in bone metabolism.

#### 4.2.2 Catabolic Action

NPY is expressed in osteoblasts, osteoclasts, osteocytes, and chondrocytes (98). NPY inhibits the differentiation and activity of osteoblast by binding to Y1 receptors on osteoblasts. The reduced bone formation and bone mass (99) would be vanished by osteoblast-specific Y1 KO (100, 101). NPY was truncated by DPP4 with a half-life of 2 to 3 min and then lost the ability of binding to the Y1 receptor (102). Thus, it is valuable to evaluate the effect of DPP4 inhibition on NPY-related bone loss.

PYY shares the same receptors with NPY and is often co-secreted with GLP-1 and GLP-2R (103). The secretion form of PYY (PYY_1−36_) containing 36–amino acid molecular was degraded by DPP4 to form PYY_3−36_ (104). PYY_1-36_ might exert suppressive effects on osteoblast activity by binding to the Y_1_ receptor (101). PYY_1-36_ was negatively associated with aBMD and P1NP (105), indicating a catabolic impact on bone metabolism. Bone mass was reduced in transgenic mouse models with overproduction of PYY, whereas bone mass and strength was increased in PYY KO mice (106). Further studies are needed to clarify whether the DPP4 degraded form PYY_3−36_ would have an anti-osteogenic effects on BMD.

### 4.3 DPP4 Regulates Immune Responses in the Bone Microenvironment

It is well known that there is a dynamic cross-talk between bone remodeling and immune systems which is called “Osteoimmunology” (107). Several diversified immune cells, including CD4^+^ T cells and CD8^+^ T cells, were found in the bone marrow (108–110). Bone cells share the same microenvironment with immune cells in the bone marrow, including cytokines, chemokines, receptors, and transcription factors. Since DPP4 was expressed on the surface of osteoblasts (111) and osteoclasts (112) and a wide range of immune cells (i.e., macrophages, lymphocytes, natural killer cells, monocytes, and dendritic cells) (113–115), it is conceivable that DPP4 would exert its effect on the “osteoimmunology”.

#### 4.3.1 T Cells

Naïve T cells played a potential protective role for bone in studies where T cell–deficient mice were presented with enhanced osteoclastogenesis and reduced BMD (116). Accordingly, activated T cells may disturb bone homeostasis and induce subsequent bone loss *via* the release of RANKL (117) and TNF-α (118) under pathological conditions such as estrogen deficiency (119, 120) and in inflammatory diseases (121). Activated T cells can be divided into cytotoxic CD8^+^ T cells and CD4^+^ T helpers cells, which is further subcategorized in Th1, Th2, Th17, and Treg cells. DPP4 is a marker of human activated T cells and an essential co-stimulatory molecule for T cells’ maturation, activation, and differentiation. It directly triggers T cell activation and proliferation by binding to ADA (30). Furthermore, DPP4 also has a costimulatory function by binding to CXCR4 (32), M6P/IGF-IIR (33), TCR/CD3 (30), CD45 (protein tyrosine phosphatase) (34), caveolin-1 (36), CARMA1 (37), and fibronectin III (38, 39). Synthetic inhibition or deficiency of DPP4 results in impaired development and maturation of naïve T cells (122–124). In addition, DPP4/CD26 plays an essential role in T cell differentiation.

Naïve CD4^+^ T cells differentiated into distinct effector T cell subsets of T helper l (Th1) and T helper 2 (Th2) (125). Both Th1 and Th2 cells are now known to inhibit osteoclast formation by secreting signature cytokines IFN-γ and IL-4, respectively (126). DPP4 is expressed at a higher level in Th1 cells but at a lower level in Th2 cells. Deleting of DPP4/CD26 in mice deregulated Th1 immune responses and reduced Th1 cytokines but upregulated Th2-type cytokines (127, 128). Accordingly, DPP4 was proposed to enhance the production of Th1 proimflamatory cytokines including IFN-γ, TNF-α, and IL-6 (115). As an osteoclastogenic subset of T cells, T helper 17 (Th17) cells secreted IFN-γ and IL-17, regulating pre-osteoclast proliferation, differentiation, and apoptosis, leading to pathological bone loss (129, 130). Treg cells (131–133) are known to have anti-osteoclastogenesis function by downregulating the expression in RANKL and macrophage colony-stimulating factor (M-CSF) (134, 135). DPP4/CD26 was highly expressed on Th17 cells (121), yet was used as a negative selection marker for Tregs. The absent/low DPP4 expression on the surface of Treg cells were important for the utilization and accumulation of adenosine to present as Treg/effector T cells (135). In addition, DPP4 inhibitors significantly increased the Treg expansion in non-obese diabetic mice (136). Hence, DPP4 may promote osteoclastogenesis by restraining proliferation and activization of Treg cells. Any imbalance within the two couple of lymphocytes (Th1, Th2, Th17, and Tregs) played a prominent role in immune regulation (137, 138). A study using solid-phase immobilized specific anti-CD3 mAb to stimulate T cells differentiation showed that the high expression of DPP4/CD26 played an indispensable role in the differentiation and functions of Th1 and Th17 lymphocytes. Thus, DPP4/CD26 could be involved in bone diseases such as osteoporosis by regulating naïve CD4^+^ T differentiation and the immune balance of its activated subsets.

CD8^+^ T cells were another kind of lymphocytes with both osteogenesis and osteoclastogenesis functions. A bi-directional regulatory loop between osteoclasts and CD25^+^FoxP3^+^CD8^+^ T cells was observed in previous researches (139). The naïve CD8^+^ T-cells primed by osteoclasts would express cytokines including FoxP3, CD25, CTLA-4, RANKL, and IFN-γ (140), which, in turn, potentially activated or suppressed osteoclast activity. The IFN-γ and CTLA-4 inhibited osteoclast activity (140, 141), whereas RANKL increased osteoclast activity. Healthy individuals taking DPP4 inhibitor sitagliptin 100 mg daily for 28 days displayed significant increase in the percentage of memory CD8^+^ T cells from days 0 to 3 compared to the placebo group (142). Therefore, these findings support further studies to verify regulation mechanisms of DPP4 on CD8^+^ T cells and clinical outcome of bi-directional regulatory loop.

#### 4.3.2 B Cells

B cells have long been recognized as active RANK/RANKL/OPG axis regulators in osteoimmunology. B cells produced OPG (116) and RANKL (143) and played dual functions on bone homeostasis. Imbalanced production cytokines of B cells between RANKL and OPG might strongly link bone turnover and the immune response. DPP4 is expressed on B cells, a natural killer (144). Reduced DNA synthesis (144, 145) in B cells and impaired immunoglobulin isotype switching of B cells were observed in DPP4-deficient mice (146). However, an *in vitro* study showed no effect of DPP4 deficiency on B cells in rats (132), whereas B cell numbers were decreased markedly in later life by monitoring the long-term effect of DPP4 deficiency *in vivo* (147). DPP4 is expressed on B cells (144); in turn, it might be involved in the proliferation and function of B cells in physiological bone remodeling.

#### 4.3.3 Macrophages

Osteomacs, a kind of bone macrophages, settles in the endosteal and periosteal surfaces of the bone (148). Loss of osteomacs leads to a complete loss of osteoblasts, which are presumed to play an essential role in maintaining mature osteoblasts (149). Macrophages are highly plastic in response to their microenvironment and could be typically divided into pro-inflammatory M1 and anti-inflammatory M2. Osteomacs cells polarization with alternatively types are important mediators in bone homeostasis (150). M1 enhances osteoclast differentiation and resorption by upregulation of RANKL, whereas M2 participated in the clearance of apoptotic cells (151). Treatment with DPP4 inhibitor Alogliptin was associated with reduced numbers of pro-inflammatory M1 macrophages and increased gene expression of M2 macrophage markers (152). Hiromura et al. (153) found that DPP4 inhibitors can induce the polarization and migration of M2 macrophages by CCL3 Caveolin-1 cell signal transcription factors in white fat. Other research (154) showed GLP-1 agonist (exenatide) would promote the polarization of M2 macrophages and the differentiation of bone marrow hematopoietic stem cells into osteoclasts through the PKA/STAT pathway. Therefore, it is speculated that DPP4, as the upper hydrolase signaling molecule of GLP-1, might regulate macrophage polarization through the GLP-1/GLP-1 receptor pathway. Accordingly, DPP4 was proposed to regulate M1/M2 polarization, resulting in a shift in macrophage phenotypes toward M1. Further studies concerning the direct role of DPP4 on osteomacs polarization would be helpful for understanding the mechanism of “osteoimmunology”.

### 4.4 DPP4 Cleaves Cytokines in Bone Microenvironment

There is increasing evidence that cytokines are critically responsible for bone resorption and formation changes. DPP4 can cleave X-Pro or X-Ala dipeptides from numerous cytokines in the bone microenvironment through its N-terminal dipeptidase activity. This cleavage can result in both inactive and active fragments of the targeted cytokines (**Table 2**). Chemokines promoted the migration of osteoclast precursor cells and facilitated the process of osteoclastogenesis and bone resorption. The NH_2_ terminal of many chemokines could be cleaved by DPP4, thus influencing their biologic activities. The widely studied CX3CL1/CX3CR1 (155), CXCL9 (156), CXCL10 (157–160), CXCL12/CXCR4 (157–160), and CCL5/CCR1 (161) signaling pathway were proved to play an important role in osteoclast formation and maturation. DPP4 removes N-terminal amino acids from their ligands or receptors and impaires its chemotactic activity (174–178). Other chemokines such as CCL2 (162, 163), CCL3 (164), CCL11 (165), and CCL20 (166) are known to have putative truncation sites for DPP4, which showed modifying effects on hematopoietic stem/progenitor cells with truncated forms (2).

**Table 2:** The effects of cytokines on bone metabolism.

Cytokines	Bone remodeling	References
**Chemokines**		
CX3CL1	Stimulation of osteoclastogenesis	(155)
CXCL9	Inhibition of osteoblast differentiation	(156)
CXCL 10	Stimulation of osteoclastogenesis and bone formation	(157–160)
CXCL 12	Stimulation of osteoclastogenesis and osteoblastogenesis	(157–160)
CCL 5	Osteoblast migration and bone formation;Inhibition of osteoclastogenesis.	(161)
CCL 2	Stimulation of osteoclastogenesis	(162, 163)
CCL 3	Stimulation of osteoclastogenesis	(164)
CCL 11	Stimulation of osteoclastogenesis and bone formation	(165)
CCL 20	Enhanced osteoblast-mediated osteoclastogenesis partly via IL-6 production	(166)
**Interleukin (IL)**		
IL-1	Stimulation of osteoclastogenesis	(167–169)
IL-6	Dual functions on Induces RANKL-dependent osteoclastogenesis	(170)
IL-10	Inhibit osteoclastogenesis	(171)
IL-17	Stimulating the production of M-CSF and RANKL in osteoblasts and MSCs, enhancing the formation of bone-resorbing osteoclasts from monocyte/macrophage precursors.	(172)
**Other cytokines**		
GM-CSF	Inhibited monocyte-derived osteoclast differentiation	(173)
G-CSF	G-CSF promoted monocyte maturation and supported differentiation of late-stage OCP cells	(167)
TNF-α	Enhances osteoclast differentiation, inhibits osteoclast apoptosis.	(168)

Some ILs, such as IL-1 (167–169), IL-6 (170), and IL-17 (172) known to enhance osteoclastogenesis or anti-osteoclastogenesis (IL-10) (171) have putative truncation site for DPP4 (173). Other cytokines, such as granulocyte macrophage (GM)–colony-stimulating factor (CSF) (173), G-CSF (167), and TNF-α (168), also take part in the regulation of immune and bone metabolism. DPP4 could induce truncated products with inactive function for GM-CSF, G-CSF (2), and TNF-α (27). The biochemical and biological functions of putative DPP4 truncation sites are poorly investigated. Previous research has shown that DPP4-cleaved substrates could get inactivated, modified receptor interactions, or modified bioactivities compared with the intact forms. For example, DPP4-cleaved GLP-1 can act through a different receptor from GLP-1R. As for PYY and NPY, the DPP4 cleavage form could enhance the selectivity of the Y2 receptor relative to the non-selective activity of the intact form. DPP4 cleavage of GIP induced an antagonistic feature for GIP-R signaling. The enzymatic activity of DPP4 has been implicated in certain disease states.

Nevertheless, the function of truncated forms of these cytokines has not been adequately appreciated. Further studies are needed to determine the functional difference between the truncated molecules and the full-length form of the protein.

## 5 Conclusion and Future Perspectives

**Figure 2:**
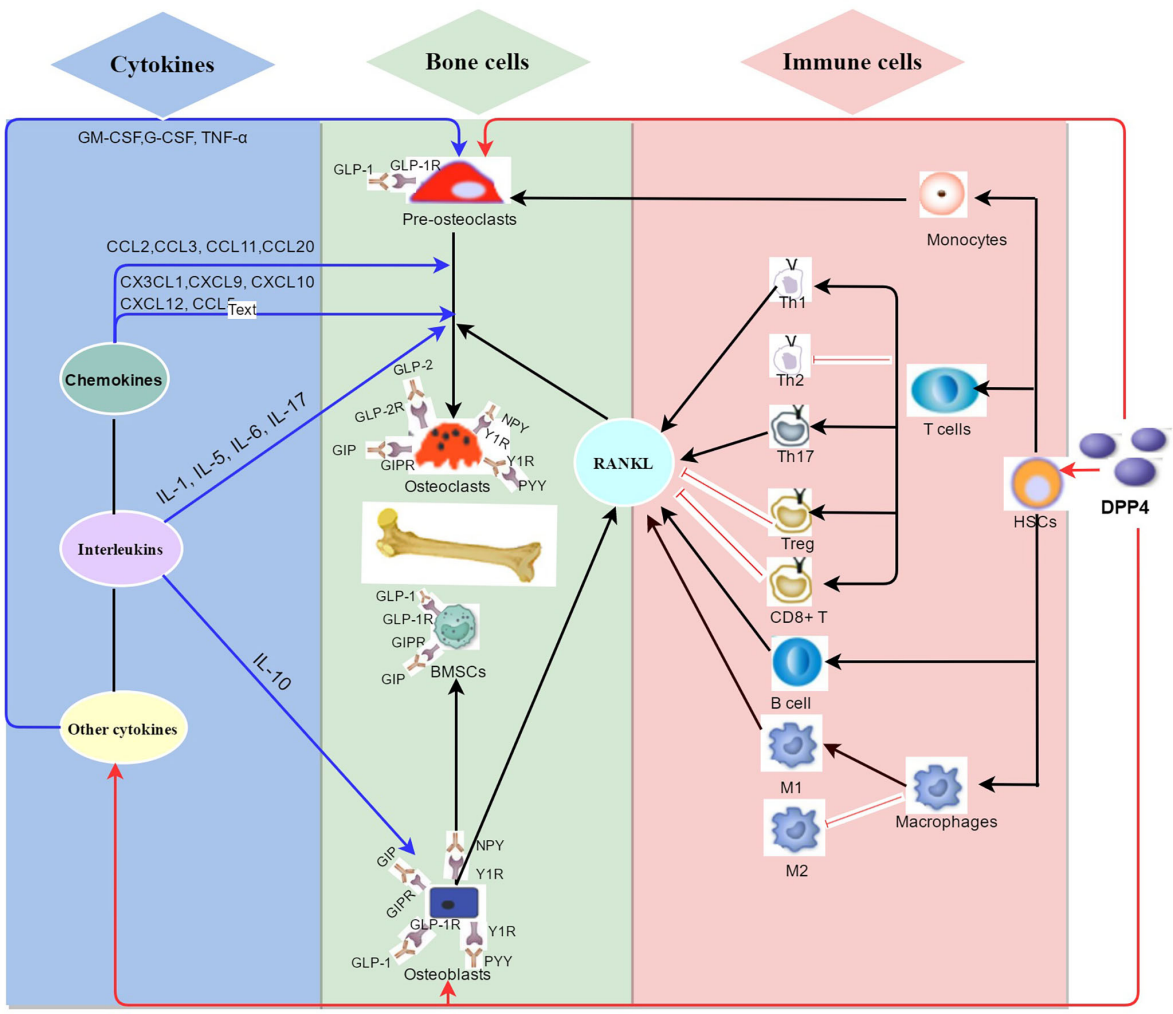
Summary of the potential mechanisms of DPP4 on bone metabolism in bone environment. As a newly discovered adipokine originated from mature adipocytes including bone marrow adipocytes, DPP4 plays a complex role in classical enzyme functions and non-enzyme functions in bone metabolism. (1) (red arrow) DPP4 might indirectly regulate bone remodeling by binding to multiple peptides substrates on bone cells such as glucagon-like peptide-1 (GLP-1), Glucagon-like peptide-2 (GLP-2), and glucose-dependent insulinotropic polypeptide (GIP), neuropeptide Y (NPY) and peptide YY (PYY). (2) (black arrow) DPP4 acts as receptor or costimulatory protein of different immunomodulation signaling process of diversified immune cells including CD4+ T cells, CD8+ T cells, B cells and macrophages. (3) (blue arrow) DPP4 hydrolyzes different sits on chemokines, interleukins, and other cytokines which take part in bone remodeling.

This review introduces the role of DPP4 on bone metabolism and summarizes its potential mechanisms (**Figure 2**). Inhibition of DPP4 activity does not directly regulate bone remodeling, whereas DPP4 indirectly affects bone metabolism by regulating DPP4 substrates and immune cells in the bone microenvironment. We speculate that increased DPP4 activity might indirectly promote bone resorption and inhibit bone formation, thereby increasing the risk of osteoporosis, showing prospects of a potentially new understanding of the mechanism of DPP4 on osteoporosis. However, the study of DPP4 on bone metabolism is still in an early stage, and there are still various questions that need to be solved. Some suggestions are purposed for future research directions:

1. It is crucial for bone remodeling to keep the energy homeostasis in the bone microenvironment. Adipokines originating from BMAT would mediate bone remodeling and energy balance through paracrine because adiponectin and leptin may be regulated by adipocytokine DPP4 in the bone microenvironment. Future studies may focus on the relationship between DPP4 and other adipocytokines in the bone microenvironment or other regulatory signals of energy metabolism.
2. Because of the extensive cross-linking signals among bone cells cytokines of bone immune cells in the bone microenvironment, it is necessary to conduct in-depth studies to find the initiating or critical mechanisms of the dialogue between DPP4 and those factors. Thus, the mechanism of DPP4 promoting bone resorption and inhibiting bone formation in bone remodeling need to be was further verified.
3. It is valuable to modify DPP4 inhibitors from the distinct regulation mechanisms of bone metabolism between DPP4 inhibitors and DPP4. So as to achieve the dual effects of lowering glucose and reducing the risk of osteoporosis.
4. Substrates cleaved by DPP4 can inactivate, modify receptor interactions, or modify biological activity. Because DPP4 has a wide range of cytokine effects on the bone microenvironment, it is necessary to investigate cytokines’ biochemical and biological functions after the DPP4 truncation site to further clarify the role of DPP4 in proteolysis.

## Author Contributions

QY,BF, and DL contributed equal to ideation and drafting and revising of the manuscript. HW and HC contributed to literature search. XC and LT revised the manuscript and contributed with intellectual ideas. XY contributed to the supervision of the study and revision of the manuscript. All authors contributed to the article and approved the submitted version.

## Funding

This work was supported by grants from the Science and Technology Department of Sichuan Province (2022YFS0136), the Sichuan University (2018SCUH0093), the 1.3.5 Project for Disciplines of Excellence, West China Hospital, Sichuan University (Nos. 2020HXFH008, ZYJC18003), and the Scientific Research Project Of Sichuan Medical Association (S20036).

## Conflict of Interest

The authors declare that the research was conducted in the absence of any commercial or financial relationships that could be construed as a potential conflict of interest.

## Publisher’s Note

All claims expressed in this article are solely those of the authors and do not necessarily represent those of their affiliated organizations, or those of the publisher, the editors and the reviewers. Any product that may be evaluated in this article, or claim that may be made by its manufacturer, is not guaranteed or endorsed by the publisher.
